# [1-Dimethyl­silyl-2-phenyl-3-(η^5^-tetra­methyl­cyclo­penta­dien­yl)­prop-1-en-1-yl-κ*C* ^1^](η^5^-penta­methyl­cyclo­penta­dien­yl)titanium(III)

**DOI:** 10.1107/S1600536809044845

**Published:** 2009-10-31

**Authors:** Martin Lamač, Anke Spannenberg, Perdita Arndt, Uwe Rosenthal

**Affiliations:** a Leibniz-Institut für Katalyse e.V. an der Universität Rostock, Albert-Einstein-Strasse 29a, 18059 Rostock, Germany

## Abstract

The title compound, [Ti(C_10_H_15_)(C_20_H_26_Si)], was obtained from the reaction of [Ti{η^5^:η^1^-C_5_Me_4_(CH_2_)}(η^5^-C_5_Me_5_)] with the alkynylsilane PhC_2_SiMe_2_H. The complex crystallizes with two independent mol­ecules in the asymmetric unit, which differ in the conformation of the propenyl unit, resulting in their having opposite helicity. No inter­molecular inter­actions or inter­actions involving the Si—H bond are present. The observed geometrical parameters are unexceptional compared to known structures of the same type.

## Related literature

For the preparation and structures of analogous compounds, see: Pinkas *et al.* (2008 ▶). For the preparation of group 4 metallocene complexes with alkynylsilanes, see: Ohff *et al.* (1995 ▶); Peulecke *et al.* (1998 ▶).
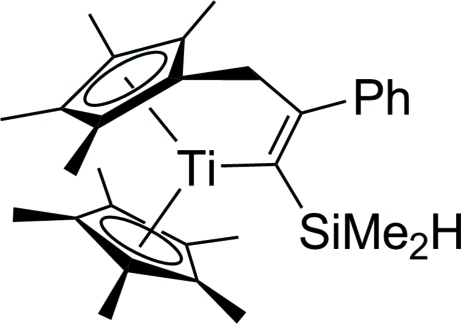


## Experimental

### 

#### Crystal data

[Ti(C_10_H_15_)(C_20_H_26_Si)]
*M* *_r_* = 477.59
Monoclinic, 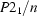

*a* = 16.4143 (3) Å
*b* = 11.6194 (3) Å
*c* = 28.0315 (5) Å
β = 94.856 (1)°
*V* = 5327.10 (19) Å^3^
*Z* = 8
Mo *K*α radiation
μ = 0.38 mm^−1^
*T* = 200 K
0.32 × 0.24 × 0.22 mm

#### Data collection

Stoe IPDS II diffractometer
Absorption correction: none
73260 measured reflections
11296 independent reflections
6527 reflections with *I* > 2σ(*I*)
*R*_int_ = 0.058

#### Refinement

*R*[*F* ^2^ > 2σ(*F* ^2^)] = 0.034
*wR*(*F* ^2^) = 0.075
*S* = 0.77
11296 reflections
607 parameters
H atoms treated by a mixture of independent and constrained refinement
Δρ_max_ = 0.26 e Å^−3^
Δρ_min_ = −0.20 e Å^−3^

### 

Data collection: *X-AREA* (Stoe & Cie, 2005 ▶); cell refinement: *X-AREA*; data reduction: *X-AREA*; program(s) used to solve structure: *SHELXS97* (Sheldrick, 2008 ▶); program(s) used to refine structure: *SHELXL97* (Sheldrick, 2008 ▶); molecular graphics: *DIAMOND* (Brandenburg, 2007 ▶); software used to prepare material for publication: *PLATON* (Spek, 2009 ▶).

## Supplementary Material

Crystal structure: contains datablocks I, global. DOI: 10.1107/S1600536809044845/rk2178sup1.cif

Structure factors: contains datablocks I. DOI: 10.1107/S1600536809044845/rk2178Isup2.hkl

Additional supplementary materials:  crystallographic information; 3D view; checkCIF report

## Figures and Tables

**Fig. 1.**
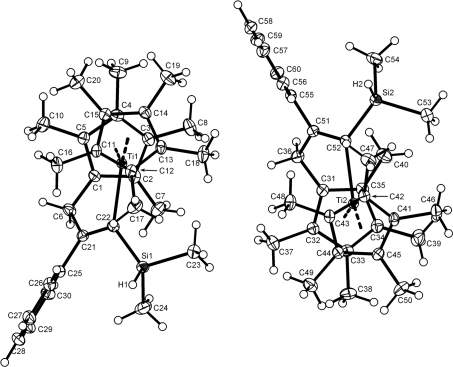
A view of the molecular structure along the crystallographic c axis showing the asymmetric unit together with the atom numbering scheme. Displacement ellipsoids are shown at 30% probability level. H atoms are presented as a small spheres of arbitrary radius.

## supplementary crystallographic information

### Comment

In order to extend our previous studies concerning the reactivity of alkynylsilanes towards Ti and Zr metallocene complexes (Ohff *et al.*, 1995; Peulecke *et al.*, 1998), we decided to explore the reaction of the tucked-in permethylated titanocene complex [Ti{η^5^:η^1^-C_5_*Me*_4_(CH_2_)}(η^5^-C_5_*Me*_5_)] with *Ph*C_2_Si*Me*_2_H. Contrary to our expectation, no reactivity involving the Si–H bond was observed. Instead, the known type of structure was obtained, which was formed by an insertion of the substituted alkyne into the titanium-methylene bond of the titanocene derivative, while the Si*Me*_2_H substituent stayed intact. Notably, only the described regioisomer was isolated as the preferentially crystallizing product of the insertion, which is in line with previous findings (Pinkas *et al.*, 2008). Observed geometrical parameters (for numbering scheme, see Fig. 1) are comparable to those of the analogous [Ti{η^5^-C_5_*Me*_4_[-CH_2_C(*Ph*)═C(Si*Me*_3_)-]} (η^5^-C_5_*Me*_5_)], while the differences between two independent molecules in the title structure are insignificant. For instance, the Ti1-C22 distance is 2.236 (2)Å (the corresponding Ti2-C52 is 2.223 (2)Å; *cf.* the distance in the reference compound: 2.251 (2)Å), the torsion angle Ti1-C22-C21-C6 is 26.3 (2)° (the corresponding Ti2-C52-C51-C36 is -26.9 (2)°; *cf.* -24.6 (2)° in the reference compound), and the torsion angle Si1-C22-C21-C25 is 31.4 (3)° (the corresponding Si2-C52-C51-C55 is -30.6 (3)°; *cf.* -29.5 (2)° in the reference compound). Ti-ring centroid distances to both substituted cyclopentadienyl rings C1-C5 and C11-C15 are 2.038 (1) and 2.072 (1)Å, respectively, the dihedral angle between least-square planes of these rings is 33.78 (12)° (2.039 (1) and 2.072 (1)Å, and 33.41 (12)° for C31-C35 and C41-C45, respectively; *cf.* the values for the reference compound: 2.047 (1) and 2.073 (1)Å, and 33.22 (5)°).

### Experimental

The title compound was obtained from the reaction of 120 mg (0.38 mmol) of [Ti{η^5^:η^1^-C_5_*Me*_4_(CH_2_)}(η^5^-C_5_*Me*_5_)] and threefold excess of *Ph*C_2_Si*Me*_2_H in 5 ml of *n*-hexane. After stirring the mixture for 3 h at room temperature the colour changed from deep purple to brown. The solvent was removed and the residue extracted with *n*-pentane. The solution afforded dark brown crystals suitable for X-ray analysis upon standing at 195 K overnight. Yield: 59 mg (33%). M.p. 406-407 K (under argon).

### Refinement

H1 and H2 were found from a difference Fourier map and refined without restraints. All other H atoms were placed in idealized positions with d(C–H) = 0.99Å (CH_2_), 0.98Å (CH_3_) and 0.95Å (CH) and refined using a riding model with *U*_iso_(H) fixed at 1.5 *U*_eq_(C) for CH_3_ and 1.2 *U*_eq_(C) for CH_2_ and CH.

### Crystal data

**Table:**

[Ti(C_10_H_15_)(C_20_H_26_Si)]	*F*(000) = 2056
*M**_r_* = 477.59	*D*_x_ = 1.191 Mg m^−^^3^
Monoclinic, *P*2_1_/*n*	Mo *K*α radiation, λ = 0.71073 Å
Hall symbol: -P 2yn	Cell parameters from 7992 reflections
*a* = 16.4143 (3) Å	θ = 1.9–27.0°
*b* = 11.6194 (3) Å	µ = 0.38 mm^−^^1^
*c* = 28.0315 (5) Å	*T* = 200 K
β = 94.856 (1)°	Prism, brown
*V* = 5327.10 (19) Å^3^	0.32 × 0.24 × 0.22 mm
*Z* = 8	

### Data collection

**Table:**

Stoe IPDS II diffractometer	6527 reflections with *I* > 2σ(*I*)
Radiation source: fine-focus sealed tube	*R*_int_ = 0.058
graphite	θ_max_ = 26.7°, θ_min_ = 1.4°
ω scans	*h* = −19→20
73260 measured reflections	*k* = −14→14
11296 independent reflections	*l* = −35→35

### Refinement

**Table:**

Refinement on *F*^2^	Primary atom site location: structure-invariant direct methods
Least-squares matrix: full	Secondary atom site location: difference Fourier map
*R*[*F*^2^ > 2σ(*F*^2^)] = 0.034	Hydrogen site location: inferred from neighbouring sites
*wR*(*F*^2^) = 0.075	H atoms treated by a mixture of independent and constrained refinement
*S* = 0.77	*w* = 1/[σ^2^(*F*_o_^2^) + (0.0382*P*)^2^] where *P* = (*F*_o_^2^ + 2*F*_c_^2^)/3
11296 reflections	(Δ/σ)_max_ = 0.001
607 parameters	Δρ_max_ = 0.26 e Å^−^^3^
0 restraints	Δρ_min_ = −0.20 e Å^−^^3^

### Special details

**Table:**

Geometry. All s.u.'s (except the s.u. in the dihedral angle between two l.s. planes) are estimated using the full covariance matrix. The cell s.u.'s are taken into account individually in the estimation of s.u.'s in distances, angles and torsion angles; correlations between s.u.'s in cell parameters are only used when they are defined by crystal symmetry. An approximate (isotropic) treatment of cell s.u.'s is used for estimating s.u.'s involving l.s. planes.
Refinement. Refinement of *F*^2^ against ALL reflections. The weighted *R*-factor *wR* and goodness of fit *S* are based on *F*^2^, conventional *R*-factors *R* are based on *F*, with *F* set to zero for negative *F*^2^. The threshold expression of *F*^2^ > σ(*F*^2^) is used only for calculating *R*-factors(gt) *etc*. and is not relevant to the choice of reflections for refinement. *R*-factors based on *F*^2^ are statistically about twice as large as those based on *F*, and *R*-factors based on ALL data will be even larger.

### Fractional atomic coordinates and isotropic or equivalent isotropic displacement parameters (Å^2^)

**Table:**

	*x*	*y*	*z*	*U*_iso_*/*U*_eq_	
C1	0.32601 (12)	0.81677 (18)	0.05676 (7)	0.0270 (4)	
C2	0.41180 (13)	0.81503 (19)	0.05322 (7)	0.0297 (5)	
C3	0.44162 (13)	0.92744 (19)	0.06261 (7)	0.0313 (5)	
C4	0.37424 (14)	0.99916 (18)	0.07003 (7)	0.0318 (5)	
C5	0.30275 (12)	0.93075 (18)	0.06759 (7)	0.0284 (5)	
C6	0.27240 (14)	0.71338 (19)	0.05791 (8)	0.0346 (5)	
H6A	0.2740	0.6698	0.0277	0.042*	
H6B	0.2153	0.7379	0.0608	0.042*	
C7	0.46226 (15)	0.7153 (2)	0.03894 (8)	0.0422 (6)	
H7A	0.4797	0.7288	0.0068	0.063*	
H7B	0.4296	0.6447	0.0389	0.063*	
H7C	0.5105	0.7073	0.0618	0.063*	
C8	0.52970 (14)	0.9631 (2)	0.06112 (9)	0.0466 (6)	
H8A	0.5624	0.9300	0.0886	0.070*	
H8B	0.5336	1.0473	0.0624	0.070*	
H8C	0.5503	0.9353	0.0314	0.070*	
C9	0.37578 (17)	1.12853 (19)	0.06992 (9)	0.0459 (6)	
H9A	0.4306	1.1554	0.0813	0.069*	
H9B	0.3360	1.1578	0.0911	0.069*	
H9C	0.3618	1.1567	0.0373	0.069*	
C10	0.21611 (13)	0.9718 (2)	0.06965 (8)	0.0420 (6)	
H10A	0.1840	0.9520	0.0397	0.063*	
H10B	0.2159	1.0555	0.0741	0.063*	
H10C	0.1921	0.9347	0.0965	0.063*	
C11	0.32990 (12)	0.89228 (18)	0.20675 (7)	0.0274 (4)	
C12	0.40412 (12)	0.83158 (18)	0.21737 (7)	0.0279 (4)	
C13	0.46852 (12)	0.90263 (19)	0.20351 (7)	0.0301 (5)	
C14	0.43409 (13)	1.00735 (18)	0.18655 (7)	0.0305 (5)	
C15	0.34842 (12)	1.00188 (18)	0.18875 (7)	0.0288 (5)	
C16	0.24569 (13)	0.8498 (2)	0.21516 (8)	0.0412 (6)	
H16A	0.2313	0.8760	0.2466	0.062*	
H16B	0.2449	0.7655	0.2141	0.062*	
H16C	0.2060	0.8802	0.1902	0.062*	
C17	0.41264 (15)	0.7241 (2)	0.24654 (8)	0.0414 (6)	
H17A	0.4639	0.6855	0.2407	0.062*	
H17B	0.3665	0.6727	0.2375	0.062*	
H17C	0.4130	0.7435	0.2806	0.062*	
C18	0.55908 (13)	0.8822 (2)	0.21251 (9)	0.0455 (6)	
H18A	0.5687	0.8086	0.2291	0.068*	
H18B	0.5837	0.9445	0.2324	0.068*	
H18C	0.5838	0.8803	0.1819	0.068*	
C19	0.48357 (15)	1.1121 (2)	0.17642 (9)	0.0461 (6)	
H19A	0.4469	1.1778	0.1698	0.069*	
H19B	0.5144	1.0975	0.1486	0.069*	
H19C	0.5217	1.1294	0.2043	0.069*	
C20	0.28851 (14)	1.0991 (2)	0.18128 (8)	0.0427 (6)	
H20A	0.2386	1.0713	0.1633	0.064*	
H20B	0.3127	1.1608	0.1633	0.064*	
H20C	0.2752	1.1287	0.2124	0.064*	
C21	0.30070 (12)	0.63626 (17)	0.10005 (7)	0.0279 (4)	
C22	0.36613 (13)	0.66434 (18)	0.13080 (7)	0.0280 (5)	
C23	0.53886 (13)	0.5882 (2)	0.16556 (9)	0.0430 (6)	
H23A	0.5719	0.5404	0.1458	0.065*	
H23B	0.5586	0.5793	0.1993	0.065*	
H23C	0.5434	0.6690	0.1562	0.065*	
C24	0.43016 (17)	0.4187 (2)	0.11409 (11)	0.0636 (8)	
H24A	0.4431	0.4469	0.0827	0.095*	
H24B	0.3763	0.3818	0.1112	0.095*	
H24C	0.4716	0.3626	0.1260	0.095*	
C25	0.25185 (12)	0.52849 (17)	0.10278 (7)	0.0284 (4)	
C26	0.22987 (13)	0.48986 (19)	0.14695 (8)	0.0349 (5)	
H26	0.2451	0.5336	0.1749	0.042*	
C27	0.18631 (14)	0.3891 (2)	0.15085 (9)	0.0416 (6)	
H27	0.1723	0.3641	0.1814	0.050*	
C28	0.16313 (14)	0.3247 (2)	0.11063 (9)	0.0442 (6)	
H28	0.1336	0.2551	0.1134	0.053*	
C29	0.18299 (15)	0.3618 (2)	0.06673 (9)	0.0443 (6)	
H29	0.1669	0.3179	0.0389	0.053*	
C30	0.22632 (14)	0.46268 (19)	0.06274 (8)	0.0370 (5)	
H30	0.2390	0.4878	0.0320	0.044*	
C31	0.81515 (12)	0.77256 (18)	0.05379 (7)	0.0283 (4)	
C32	0.79439 (13)	0.65761 (18)	0.06447 (7)	0.0283 (5)	
C33	0.86659 (13)	0.59103 (18)	0.06494 (7)	0.0307 (5)	
C34	0.93196 (13)	0.66513 (19)	0.05530 (7)	0.0324 (5)	
C35	0.90001 (13)	0.77657 (18)	0.04733 (7)	0.0298 (5)	
C36	0.76127 (13)	0.87527 (18)	0.05787 (7)	0.0335 (5)	
H36A	0.7053	0.8498	0.0634	0.040*	
H36B	0.7584	0.9197	0.0276	0.040*	
C37	0.70856 (13)	0.6143 (2)	0.06782 (8)	0.0414 (6)	
H37A	0.6772	0.6232	0.0367	0.062*	
H37B	0.6825	0.6586	0.0921	0.062*	
H37C	0.7102	0.5328	0.0769	0.062*	
C38	0.86991 (16)	0.46165 (18)	0.06501 (8)	0.0421 (6)	
H38A	0.8576	0.4330	0.0323	0.063*	
H38B	0.8296	0.4314	0.0856	0.063*	
H38C	0.9247	0.4363	0.0772	0.063*	
C39	1.01891 (14)	0.6297 (2)	0.04940 (9)	0.0505 (6)	
H39A	1.0261	0.6195	0.0153	0.076*	
H39B	1.0308	0.5571	0.0663	0.076*	
H39C	1.0563	0.6895	0.0627	0.076*	
C40	0.94667 (15)	0.8778 (2)	0.03055 (8)	0.0432 (6)	
H40A	0.9990	0.8844	0.0500	0.065*	
H40B	0.9147	0.9482	0.0338	0.065*	
H40C	0.9568	0.8669	−0.0031	0.065*	
C41	0.96798 (12)	0.68581 (19)	0.19751 (7)	0.0295 (5)	
C42	0.90395 (12)	0.75580 (17)	0.21228 (7)	0.0270 (4)	
C43	0.82958 (12)	0.69442 (18)	0.20265 (7)	0.0276 (4)	
C44	0.84810 (12)	0.58542 (18)	0.18456 (7)	0.0286 (4)	
C45	0.93381 (12)	0.58074 (18)	0.18101 (7)	0.0304 (5)	
C46	1.05862 (13)	0.7072 (2)	0.20591 (9)	0.0431 (6)	
H46A	1.0844	0.6434	0.2244	0.065*	
H46B	1.0681	0.7792	0.2238	0.065*	
H46C	1.0823	0.7131	0.1750	0.065*	
C47	0.91308 (15)	0.86374 (19)	0.24154 (8)	0.0393 (5)	
H47A	0.9170	0.8440	0.2757	0.059*	
H47B	0.8655	0.9134	0.2340	0.059*	
H47C	0.9628	0.9044	0.2341	0.059*	
C48	0.74549 (13)	0.7364 (2)	0.21174 (8)	0.0391 (5)	
H48A	0.7068	0.7164	0.1844	0.059*	
H48B	0.7466	0.8202	0.2159	0.059*	
H48C	0.7283	0.7000	0.2408	0.059*	
C49	0.78849 (14)	0.4877 (2)	0.17790 (8)	0.0418 (6)	
H49A	0.7747	0.4602	0.2093	0.063*	
H49B	0.8131	0.4248	0.1608	0.063*	
H49C	0.7388	0.5142	0.1593	0.063*	
C50	0.98355 (15)	0.4770 (2)	0.16986 (9)	0.0438 (6)	
H50A	1.0157	0.4943	0.1428	0.066*	
H50B	0.9469	0.4121	0.1615	0.066*	
H50C	1.0205	0.4569	0.1979	0.066*	
C51	0.79481 (12)	0.95176 (17)	0.09929 (7)	0.0278 (4)	
C52	0.86394 (11)	0.92305 (17)	0.12721 (7)	0.0256 (4)	
C53	1.03847 (14)	0.9989 (2)	0.15754 (10)	0.0466 (6)	
H53A	1.0443	0.9304	0.1780	0.070*	
H53B	1.0726	1.0611	0.1719	0.070*	
H53C	1.0558	0.9806	0.1258	0.070*	
C54	0.92600 (16)	1.1694 (2)	0.10873 (10)	0.0546 (7)	
H54A	0.9346	1.1409	0.0766	0.082*	
H54B	0.9692	1.2244	0.1190	0.082*	
H54C	0.8727	1.2075	0.1081	0.082*	
C55	0.74705 (12)	1.05918 (17)	0.10507 (7)	0.0272 (4)	
C56	0.73006 (13)	1.09480 (19)	0.15038 (8)	0.0339 (5)	
H56	0.7478	1.0490	0.1773	0.041*	
C57	0.68790 (14)	1.1954 (2)	0.15717 (9)	0.0415 (6)	
H57	0.6772	1.2182	0.1886	0.050*	
C58	0.66136 (13)	1.2626 (2)	0.11861 (9)	0.0413 (5)	
H58	0.6332	1.3326	0.1233	0.050*	
C59	0.67563 (14)	1.22821 (19)	0.07342 (9)	0.0404 (5)	
H59	0.6568	1.2741	0.0467	0.048*	
C60	0.71741 (13)	1.12665 (19)	0.06637 (8)	0.0359 (5)	
H60	0.7259	1.1028	0.0347	0.043*	
Si1	0.42936 (4)	0.54183 (5)	0.15673 (2)	0.03421 (15)	
Si2	0.92888 (4)	1.04570 (5)	0.15165 (2)	0.03194 (14)	
Ti1	0.38431 (2)	0.85502 (3)	0.132787 (13)	0.02321 (9)	
Ti2	0.88083 (2)	0.73319 (3)	0.127974 (13)	0.02302 (9)	
H1	0.4075 (13)	0.4888 (19)	0.2016 (8)	0.045 (6)*	
H2	0.9127 (12)	1.0935 (18)	0.1958 (7)	0.036 (6)*	

### Atomic displacement parameters (Å^2^)

**Table:**

	*U*^11^	*U*^22^	*U*^33^	*U*^12^	*U*^13^	*U*^23^
C1	0.0293 (11)	0.0286 (11)	0.0223 (10)	−0.0043 (9)	−0.0031 (8)	0.0030 (8)
C2	0.0291 (11)	0.0376 (12)	0.0225 (10)	0.0025 (9)	0.0018 (8)	0.0011 (9)
C3	0.0315 (11)	0.0372 (12)	0.0254 (11)	−0.0055 (9)	0.0042 (9)	0.0023 (9)
C4	0.0388 (12)	0.0302 (12)	0.0262 (11)	−0.0026 (10)	0.0013 (9)	0.0027 (9)
C5	0.0287 (11)	0.0330 (12)	0.0230 (10)	0.0027 (9)	−0.0003 (8)	0.0041 (8)
C6	0.0350 (12)	0.0334 (13)	0.0335 (12)	−0.0078 (10)	−0.0087 (9)	0.0029 (9)
C7	0.0442 (14)	0.0468 (14)	0.0365 (13)	0.0108 (11)	0.0076 (10)	−0.0016 (10)
C8	0.0363 (13)	0.0624 (17)	0.0419 (14)	−0.0150 (12)	0.0078 (10)	0.0035 (12)
C9	0.0645 (16)	0.0312 (13)	0.0414 (14)	−0.0052 (12)	0.0012 (12)	0.0060 (10)
C10	0.0369 (13)	0.0502 (15)	0.0381 (13)	0.0089 (11)	−0.0014 (10)	0.0061 (11)
C11	0.0220 (10)	0.0352 (12)	0.0252 (10)	0.0004 (9)	0.0037 (8)	−0.0004 (9)
C12	0.0269 (10)	0.0334 (11)	0.0234 (10)	0.0032 (9)	0.0023 (8)	−0.0017 (9)
C13	0.0235 (10)	0.0405 (13)	0.0254 (11)	−0.0007 (9)	−0.0025 (8)	−0.0061 (9)
C14	0.0326 (11)	0.0327 (12)	0.0263 (11)	−0.0059 (9)	0.0033 (9)	−0.0063 (9)
C15	0.0291 (11)	0.0327 (11)	0.0243 (11)	0.0028 (9)	0.0010 (8)	−0.0032 (8)
C16	0.0282 (12)	0.0562 (16)	0.0398 (13)	−0.0029 (11)	0.0061 (10)	0.0020 (11)
C17	0.0487 (14)	0.0438 (14)	0.0321 (12)	0.0124 (11)	0.0064 (10)	0.0067 (10)
C18	0.0256 (11)	0.0653 (17)	0.0448 (14)	0.0029 (11)	−0.0026 (10)	−0.0116 (12)
C19	0.0504 (15)	0.0453 (15)	0.0428 (14)	−0.0189 (12)	0.0044 (11)	−0.0075 (11)
C20	0.0459 (14)	0.0398 (14)	0.0420 (14)	0.0133 (11)	0.0021 (11)	−0.0030 (11)
C21	0.0284 (10)	0.0271 (11)	0.0280 (11)	0.0009 (9)	0.0019 (8)	−0.0007 (9)
C22	0.0304 (12)	0.0266 (11)	0.0266 (10)	−0.0026 (9)	0.0005 (9)	0.0012 (9)
C23	0.0356 (13)	0.0408 (14)	0.0521 (15)	0.0009 (11)	0.0008 (11)	0.0021 (11)
C24	0.0584 (17)	0.0354 (15)	0.093 (2)	0.0135 (13)	−0.0164 (16)	−0.0120 (14)
C25	0.0251 (10)	0.0290 (11)	0.0307 (11)	0.0002 (9)	−0.0007 (8)	−0.0002 (9)
C26	0.0315 (12)	0.0383 (13)	0.0347 (12)	−0.0015 (10)	0.0018 (9)	−0.0013 (10)
C27	0.0350 (13)	0.0415 (14)	0.0490 (14)	−0.0046 (11)	0.0070 (10)	0.0085 (11)
C28	0.0352 (13)	0.0326 (13)	0.0636 (17)	−0.0097 (11)	−0.0030 (11)	0.0043 (12)
C29	0.0484 (14)	0.0345 (13)	0.0480 (15)	−0.0098 (11)	−0.0077 (11)	−0.0071 (11)
C30	0.0423 (13)	0.0341 (13)	0.0339 (12)	−0.0053 (10)	−0.0011 (10)	−0.0029 (10)
C31	0.0304 (11)	0.0321 (11)	0.0216 (10)	0.0012 (9)	−0.0017 (8)	−0.0037 (9)
C32	0.0306 (11)	0.0312 (12)	0.0225 (10)	−0.0025 (9)	−0.0011 (8)	−0.0053 (9)
C33	0.0365 (12)	0.0292 (12)	0.0264 (11)	0.0019 (10)	0.0029 (9)	−0.0049 (9)
C34	0.0316 (11)	0.0385 (13)	0.0278 (11)	0.0018 (10)	0.0069 (9)	−0.0067 (9)
C35	0.0339 (11)	0.0327 (11)	0.0230 (10)	−0.0024 (9)	0.0038 (8)	−0.0025 (9)
C36	0.0353 (12)	0.0332 (12)	0.0304 (12)	0.0030 (10)	−0.0069 (9)	−0.0020 (9)
C37	0.0352 (12)	0.0453 (14)	0.0431 (14)	−0.0089 (11)	−0.0011 (10)	−0.0035 (11)
C38	0.0586 (16)	0.0280 (13)	0.0392 (13)	0.0043 (11)	0.0015 (11)	−0.0069 (10)
C39	0.0396 (13)	0.0594 (16)	0.0548 (16)	0.0079 (12)	0.0167 (11)	−0.0083 (13)
C40	0.0507 (14)	0.0444 (14)	0.0359 (13)	−0.0095 (11)	0.0109 (11)	0.0020 (10)
C41	0.0218 (10)	0.0389 (12)	0.0274 (11)	−0.0005 (9)	−0.0011 (8)	0.0080 (9)
C42	0.0279 (10)	0.0310 (11)	0.0215 (10)	−0.0007 (9)	−0.0007 (8)	0.0003 (8)
C43	0.0239 (10)	0.0360 (12)	0.0230 (10)	0.0006 (9)	0.0019 (8)	0.0040 (8)
C44	0.0280 (11)	0.0310 (11)	0.0264 (11)	−0.0026 (9)	−0.0006 (8)	0.0038 (9)
C45	0.0303 (11)	0.0321 (12)	0.0285 (11)	0.0048 (9)	0.0007 (9)	0.0024 (9)
C46	0.0243 (11)	0.0563 (16)	0.0480 (14)	−0.0037 (11)	−0.0017 (10)	0.0070 (11)
C47	0.0493 (14)	0.0395 (13)	0.0292 (12)	−0.0080 (11)	0.0035 (10)	−0.0045 (10)
C48	0.0276 (11)	0.0537 (15)	0.0370 (12)	0.0021 (10)	0.0078 (9)	−0.0018 (11)
C49	0.0442 (14)	0.0383 (13)	0.0425 (14)	−0.0100 (11)	0.0012 (11)	0.0054 (10)
C50	0.0477 (14)	0.0402 (14)	0.0429 (14)	0.0175 (11)	0.0002 (11)	0.0035 (11)
C51	0.0300 (11)	0.0281 (11)	0.0253 (11)	0.0012 (9)	0.0023 (8)	0.0016 (8)
C52	0.0259 (10)	0.0251 (10)	0.0256 (10)	−0.0009 (8)	0.0018 (8)	0.0003 (8)
C53	0.0323 (13)	0.0420 (14)	0.0654 (17)	−0.0045 (11)	0.0033 (11)	−0.0008 (12)
C54	0.0531 (16)	0.0321 (14)	0.0766 (19)	−0.0104 (12)	−0.0056 (14)	0.0097 (13)
C55	0.0245 (10)	0.0276 (11)	0.0294 (11)	−0.0008 (8)	0.0003 (8)	−0.0011 (8)
C56	0.0318 (11)	0.0375 (13)	0.0324 (12)	0.0039 (10)	0.0020 (9)	−0.0012 (9)
C57	0.0361 (13)	0.0447 (14)	0.0441 (14)	0.0056 (11)	0.0053 (10)	−0.0124 (11)
C58	0.0308 (12)	0.0319 (12)	0.0604 (16)	0.0070 (10)	−0.0005 (11)	−0.0063 (11)
C59	0.0403 (13)	0.0332 (12)	0.0459 (14)	0.0053 (10)	−0.0064 (11)	0.0071 (10)
C60	0.0391 (12)	0.0353 (13)	0.0328 (12)	0.0054 (10)	0.0009 (9)	0.0009 (9)
Si1	0.0327 (3)	0.0266 (3)	0.0419 (4)	−0.0004 (3)	−0.0056 (3)	0.0060 (3)
Si2	0.0295 (3)	0.0264 (3)	0.0391 (4)	−0.0005 (3)	−0.0017 (3)	−0.0047 (3)
Ti1	0.02236 (18)	0.02384 (19)	0.02319 (18)	−0.00113 (15)	0.00053 (14)	0.00132 (15)
Ti2	0.02240 (19)	0.02309 (19)	0.02351 (19)	0.00067 (16)	0.00162 (14)	−0.00133 (15)

### Geometric parameters (Å, °)

**Table:**

C1—C5	1.418 (3)	C31—C35	1.421 (3)
C1—C2	1.420 (3)	C31—C36	1.496 (3)
C1—C6	1.491 (3)	C31—Ti2	2.306 (2)
C1—Ti1	2.3044 (19)	C32—C33	1.414 (3)
C2—C3	1.412 (3)	C32—C37	1.507 (3)
C2—C7	1.498 (3)	C32—Ti2	2.351 (2)
C2—Ti1	2.359 (2)	C33—C34	1.419 (3)
C3—C4	1.414 (3)	C33—C38	1.504 (3)
C3—C8	1.508 (3)	C33—Ti2	2.416 (2)
C3—Ti1	2.403 (2)	C34—C35	1.408 (3)
C4—C5	1.414 (3)	C34—C39	1.508 (3)
C4—C9	1.503 (3)	C34—Ti2	2.402 (2)
C4—Ti1	2.425 (2)	C35—C40	1.501 (3)
C5—C10	1.506 (3)	C35—Ti2	2.3629 (19)
C5—Ti1	2.344 (2)	C36—C51	1.527 (3)
C6—C21	1.524 (3)	C36—H36A	0.9900
C6—H6A	0.9900	C36—H36B	0.9900
C6—H6B	0.9900	C37—H37A	0.9800
C7—H7A	0.9800	C37—H37B	0.9800
C7—H7B	0.9800	C37—H37C	0.9800
C7—H7C	0.9800	C38—H38A	0.9800
C8—H8A	0.9800	C38—H38B	0.9800
C8—H8B	0.9800	C38—H38C	0.9800
C8—H8C	0.9800	C39—H39A	0.9800
C9—H9A	0.9800	C39—H39B	0.9800
C9—H9B	0.9800	C39—H39C	0.9800
C9—H9C	0.9800	C40—H40A	0.9800
C10—H10A	0.9800	C40—H40B	0.9800
C10—H10B	0.9800	C40—H40C	0.9800
C10—H10C	0.9800	C41—C45	1.405 (3)
C11—C15	1.412 (3)	C41—C42	1.418 (3)
C11—C12	1.417 (3)	C41—C46	1.507 (3)
C11—C16	1.505 (3)	C41—Ti2	2.383 (2)
C11—Ti1	2.3658 (19)	C42—C43	1.420 (3)
C12—C13	1.421 (3)	C42—C47	1.499 (3)
C12—C17	1.493 (3)	C42—Ti2	2.3762 (19)
C12—Ti1	2.381 (2)	C43—C44	1.407 (3)
C13—C14	1.407 (3)	C43—C48	1.506 (3)
C13—C18	1.505 (3)	C43—Ti2	2.3645 (19)
C13—Ti1	2.384 (2)	C44—C45	1.420 (3)
C14—C15	1.414 (3)	C44—C49	1.500 (3)
C14—C19	1.504 (3)	C44—Ti2	2.428 (2)
C14—Ti1	2.421 (2)	C45—C50	1.504 (3)
C15—C20	1.500 (3)	C45—Ti2	2.426 (2)
C15—Ti1	2.424 (2)	C46—H46A	0.9800
C16—H16A	0.9800	C46—H46B	0.9800
C16—H16B	0.9800	C46—H46C	0.9800
C16—H16C	0.9800	C47—H47A	0.9800
C17—H17A	0.9800	C47—H47B	0.9800
C17—H17B	0.9800	C47—H47C	0.9800
C17—H17C	0.9800	C48—H48A	0.9800
C18—H18A	0.9800	C48—H48B	0.9800
C18—H18B	0.9800	C48—H48C	0.9800
C18—H18C	0.9800	C49—H49A	0.9800
C19—H19A	0.9800	C49—H49B	0.9800
C19—H19B	0.9800	C49—H49C	0.9800
C19—H19C	0.9800	C50—H50A	0.9800
C20—H20A	0.9800	C50—H50B	0.9800
C20—H20B	0.9800	C50—H50C	0.9800
C20—H20C	0.9800	C51—C52	1.364 (3)
C21—C22	1.359 (3)	C51—C55	1.490 (3)
C21—C25	1.492 (3)	C52—Si2	1.874 (2)
C22—Si1	1.872 (2)	C52—Ti2	2.223 (2)
C22—Ti1	2.236 (2)	C53—Si2	1.873 (2)
C23—Si1	1.872 (2)	C53—H53A	0.9800
C23—H23A	0.9800	C53—H53B	0.9800
C23—H23B	0.9800	C53—H53C	0.9800
C23—H23C	0.9800	C54—Si2	1.872 (2)
C24—Si1	1.865 (3)	C54—H54A	0.9800
C24—H24A	0.9800	C54—H54B	0.9800
C24—H24B	0.9800	C54—H54C	0.9800
C24—H24C	0.9800	C55—C56	1.386 (3)
C25—C30	1.393 (3)	C55—C60	1.392 (3)
C25—C26	1.393 (3)	C56—C57	1.379 (3)
C26—C27	1.381 (3)	C56—H56	0.9500
C26—H26	0.9500	C57—C58	1.374 (3)
C27—C28	1.380 (3)	C57—H57	0.9500
C27—H27	0.9500	C58—C59	1.367 (3)
C28—C29	1.369 (3)	C58—H58	0.9500
C28—H28	0.9500	C59—C60	1.387 (3)
C29—C30	1.381 (3)	C59—H59	0.9500
C29—H29	0.9500	C60—H60	0.9500
C30—H30	0.9500	Si1—H1	1.47 (2)
C31—C32	1.417 (3)	Si2—H2	1.40 (2)
			
C5—C1—C2	108.35 (19)	C45—C41—C46	123.8 (2)
C5—C1—C6	125.28 (19)	C42—C41—C46	127.3 (2)
C2—C1—C6	125.5 (2)	C45—C41—Ti2	74.71 (12)
C5—C1—Ti1	73.75 (11)	C42—C41—Ti2	72.41 (11)
C2—C1—Ti1	74.38 (11)	C46—C41—Ti2	127.29 (14)
C6—C1—Ti1	109.57 (13)	C41—C42—C43	107.63 (18)
C3—C2—C1	107.69 (19)	C41—C42—C47	126.67 (19)
C3—C2—C7	125.1 (2)	C43—C42—C47	124.67 (19)
C1—C2—C7	127.1 (2)	C41—C42—Ti2	72.91 (11)
C3—C2—Ti1	74.48 (11)	C43—C42—Ti2	72.12 (11)
C1—C2—Ti1	70.18 (11)	C47—C42—Ti2	129.55 (14)
C7—C2—Ti1	124.01 (14)	C44—C43—C42	108.19 (17)
C2—C3—C4	108.03 (18)	C44—C43—C48	125.57 (19)
C2—C3—C8	124.6 (2)	C42—C43—C48	126.21 (19)
C4—C3—C8	127.3 (2)	C44—C43—Ti2	75.44 (11)
C2—C3—Ti1	71.04 (11)	C42—C43—Ti2	73.03 (11)
C4—C3—Ti1	73.79 (11)	C48—C43—Ti2	119.20 (14)
C8—C3—Ti1	124.16 (15)	C43—C44—C45	107.77 (18)
C5—C4—C3	108.52 (19)	C43—C44—C49	124.59 (19)
C5—C4—C9	125.2 (2)	C45—C44—C49	127.0 (2)
C3—C4—C9	125.1 (2)	C43—C44—Ti2	70.45 (11)
C5—C4—Ti1	69.64 (11)	C45—C44—Ti2	72.90 (11)
C3—C4—Ti1	72.15 (12)	C49—C44—Ti2	129.19 (14)
C9—C4—Ti1	133.79 (15)	C41—C45—C44	108.29 (18)
C4—C5—C1	107.33 (18)	C41—C45—C50	123.80 (19)
C4—C5—C10	127.0 (2)	C44—C45—C50	127.2 (2)
C1—C5—C10	125.2 (2)	C41—C45—Ti2	71.32 (12)
C4—C5—Ti1	75.90 (12)	C44—C45—Ti2	73.09 (12)
C1—C5—Ti1	70.73 (11)	C50—C45—Ti2	129.10 (14)
C10—C5—Ti1	124.61 (14)	C41—C46—H46A	109.5
C1—C6—C21	110.42 (17)	C41—C46—H46B	109.5
C1—C6—H6A	109.6	H46A—C46—H46B	109.5
C21—C6—H6A	109.6	C41—C46—H46C	109.5
C1—C6—H6B	109.6	H46A—C46—H46C	109.5
C21—C6—H6B	109.6	H46B—C46—H46C	109.5
H6A—C6—H6B	108.1	C42—C47—H47A	109.5
C2—C7—H7A	109.5	C42—C47—H47B	109.5
C2—C7—H7B	109.5	H47A—C47—H47B	109.5
H7A—C7—H7B	109.5	C42—C47—H47C	109.5
C2—C7—H7C	109.5	H47A—C47—H47C	109.5
H7A—C7—H7C	109.5	H47B—C47—H47C	109.5
H7B—C7—H7C	109.5	C43—C48—H48A	109.5
C3—C8—H8A	109.5	C43—C48—H48B	109.5
C3—C8—H8B	109.5	H48A—C48—H48B	109.5
H8A—C8—H8B	109.5	C43—C48—H48C	109.5
C3—C8—H8C	109.5	H48A—C48—H48C	109.5
H8A—C8—H8C	109.5	H48B—C48—H48C	109.5
H8B—C8—H8C	109.5	C44—C49—H49A	109.5
C4—C9—H9A	109.5	C44—C49—H49B	109.5
C4—C9—H9B	109.5	H49A—C49—H49B	109.5
H9A—C9—H9B	109.5	C44—C49—H49C	109.5
C4—C9—H9C	109.5	H49A—C49—H49C	109.5
H9A—C9—H9C	109.5	H49B—C49—H49C	109.5
H9B—C9—H9C	109.5	C45—C50—H50A	109.5
C5—C10—H10A	109.5	C45—C50—H50B	109.5
C5—C10—H10B	109.5	H50A—C50—H50B	109.5
H10A—C10—H10B	109.5	C45—C50—H50C	109.5
C5—C10—H10C	109.5	H50A—C50—H50C	109.5
H10A—C10—H10C	109.5	H50B—C50—H50C	109.5
H10B—C10—H10C	109.5	C52—C51—C55	124.22 (18)
C15—C11—C12	108.46 (17)	C52—C51—C36	121.50 (18)
C15—C11—C16	125.45 (19)	C55—C51—C36	114.26 (17)
C12—C11—C16	126.04 (19)	C51—C52—Si2	116.32 (15)
C15—C11—Ti1	75.12 (11)	C51—C52—Ti2	110.20 (14)
C12—C11—Ti1	73.24 (11)	Si2—C52—Ti2	133.18 (10)
C16—C11—Ti1	119.81 (14)	Si2—C53—H53A	109.5
C11—C12—C13	107.43 (18)	Si2—C53—H53B	109.5
C11—C12—C17	124.78 (18)	H53A—C53—H53B	109.5
C13—C12—C17	126.75 (19)	Si2—C53—H53C	109.5
C11—C12—Ti1	72.02 (11)	H53A—C53—H53C	109.5
C13—C12—Ti1	72.74 (11)	H53B—C53—H53C	109.5
C17—C12—Ti1	129.76 (15)	Si2—C54—H54A	109.5
C14—C13—C12	107.93 (17)	Si2—C54—H54B	109.5
C14—C13—C18	123.7 (2)	H54A—C54—H54B	109.5
C12—C13—C18	127.6 (2)	Si2—C54—H54C	109.5
C14—C13—Ti1	74.43 (12)	H54A—C54—H54C	109.5
C12—C13—Ti1	72.56 (11)	H54B—C54—H54C	109.5
C18—C13—Ti1	126.71 (14)	C56—C55—C60	117.48 (19)
C13—C14—C15	108.59 (18)	C56—C55—C51	119.87 (18)
C13—C14—C19	123.8 (2)	C60—C55—C51	122.64 (18)
C15—C14—C19	126.8 (2)	C57—C56—C55	121.4 (2)
C13—C14—Ti1	71.52 (12)	C57—C56—H56	119.3
C15—C14—Ti1	73.13 (12)	C55—C56—H56	119.3
C19—C14—Ti1	129.31 (14)	C58—C57—C56	120.1 (2)
C11—C15—C14	107.51 (18)	C58—C57—H57	119.9
C11—C15—C20	124.77 (19)	C56—C57—H57	119.9
C14—C15—C20	127.0 (2)	C59—C58—C57	119.7 (2)
C11—C15—Ti1	70.60 (11)	C59—C58—H58	120.2
C14—C15—Ti1	72.93 (11)	C57—C58—H58	120.2
C20—C15—Ti1	129.19 (14)	C58—C59—C60	120.4 (2)
C11—C16—H16A	109.5	C58—C59—H59	119.8
C11—C16—H16B	109.5	C60—C59—H59	119.8
H16A—C16—H16B	109.5	C59—C60—C55	120.8 (2)
C11—C16—H16C	109.5	C59—C60—H60	119.6
H16A—C16—H16C	109.5	C55—C60—H60	119.6
H16B—C16—H16C	109.5	C24—Si1—C22	111.61 (11)
C12—C17—H17A	109.5	C24—Si1—C23	104.27 (12)
C12—C17—H17B	109.5	C22—Si1—C23	109.10 (10)
H17A—C17—H17B	109.5	C24—Si1—H1	104.0 (9)
C12—C17—H17C	109.5	C22—Si1—H1	118.9 (9)
H17A—C17—H17C	109.5	C23—Si1—H1	108.0 (9)
H17B—C17—H17C	109.5	C54—Si2—C53	104.57 (12)
C13—C18—H18A	109.5	C54—Si2—C52	111.48 (11)
C13—C18—H18B	109.5	C53—Si2—C52	108.88 (10)
H18A—C18—H18B	109.5	C54—Si2—H2	105.5 (8)
C13—C18—H18C	109.5	C53—Si2—H2	106.8 (8)
H18A—C18—H18C	109.5	C52—Si2—H2	118.6 (8)
H18B—C18—H18C	109.5	C22—Ti1—C1	75.03 (7)
C14—C19—H19A	109.5	C22—Ti1—C5	106.64 (8)
C14—C19—H19B	109.5	C1—Ti1—C5	35.52 (7)
H19A—C19—H19B	109.5	C22—Ti1—C2	79.45 (8)
C14—C19—H19C	109.5	C1—Ti1—C2	35.44 (7)
H19A—C19—H19C	109.5	C5—Ti1—C2	58.60 (7)
H19B—C19—H19C	109.5	C22—Ti1—C11	98.26 (7)
C15—C20—H20A	109.5	C1—Ti1—C11	133.46 (7)
C15—C20—H20B	109.5	C5—Ti1—C11	112.43 (7)
H20A—C20—H20B	109.5	C2—Ti1—C11	168.89 (7)
C15—C20—H20C	109.5	C22—Ti1—C12	85.31 (7)
H20A—C20—H20C	109.5	C1—Ti1—C12	155.48 (7)
H20B—C20—H20C	109.5	C5—Ti1—C12	147.16 (7)
C22—C21—C25	124.62 (18)	C2—Ti1—C12	153.89 (7)
C22—C21—C6	121.56 (18)	C11—Ti1—C12	34.74 (7)
C25—C21—C6	113.80 (17)	C22—Ti1—C13	108.51 (8)
C21—C22—Si1	116.51 (16)	C1—Ti1—C13	168.53 (7)
C21—C22—Ti1	110.52 (15)	C5—Ti1—C13	144.50 (8)
Si1—C22—Ti1	132.49 (11)	C2—Ti1—C13	133.48 (7)
Si1—C23—H23A	109.5	C11—Ti1—C13	57.59 (7)
Si1—C23—H23B	109.5	C12—Ti1—C13	34.70 (7)
H23A—C23—H23B	109.5	C22—Ti1—C3	112.78 (7)
Si1—C23—H23C	109.5	C1—Ti1—C3	58.07 (7)
H23A—C23—H23C	109.5	C5—Ti1—C3	57.83 (7)
H23B—C23—H23C	109.5	C2—Ti1—C3	34.47 (7)
Si1—C24—H24A	109.5	C11—Ti1—C3	148.89 (7)
Si1—C24—H24B	109.5	C12—Ti1—C3	145.60 (7)
H24A—C24—H24B	109.5	C13—Ti1—C3	111.09 (7)
Si1—C24—H24C	109.5	C22—Ti1—C14	141.01 (7)
H24A—C24—H24C	109.5	C1—Ti1—C14	143.94 (7)
H24B—C24—H24C	109.5	C5—Ti1—C14	110.57 (7)
C30—C25—C26	117.16 (19)	C2—Ti1—C14	130.62 (7)
C30—C25—C21	123.06 (19)	C11—Ti1—C14	56.86 (7)
C26—C25—C21	119.78 (18)	C12—Ti1—C14	56.87 (7)
C27—C26—C25	121.2 (2)	C13—Ti1—C14	34.05 (7)
C27—C26—H26	119.4	C3—Ti1—C14	96.81 (7)
C25—C26—H26	119.4	C22—Ti1—C15	132.42 (7)
C28—C27—C26	120.3 (2)	C1—Ti1—C15	129.02 (7)
C28—C27—H27	119.9	C5—Ti1—C15	94.95 (7)
C26—C27—H27	119.9	C2—Ti1—C15	145.79 (7)
C29—C28—C27	119.6 (2)	C11—Ti1—C15	34.27 (7)
C29—C28—H28	120.2	C12—Ti1—C15	57.07 (7)
C27—C28—H28	120.2	C13—Ti1—C15	56.91 (7)
C28—C29—C30	120.2 (2)	C3—Ti1—C15	114.62 (7)
C28—C29—H29	119.9	C14—Ti1—C15	33.94 (7)
C30—C29—H29	119.9	C22—Ti1—C4	131.74 (7)
C29—C30—C25	121.5 (2)	C1—Ti1—C4	57.63 (7)
C29—C30—H30	119.2	C5—Ti1—C4	34.45 (7)
C25—C30—H30	119.2	C2—Ti1—C4	57.10 (7)
C32—C31—C35	108.29 (19)	C11—Ti1—C4	120.12 (7)
C32—C31—C36	125.58 (19)	C12—Ti1—C4	142.81 (7)
C35—C31—C36	125.18 (19)	C13—Ti1—C4	116.25 (7)
C32—C31—Ti2	74.03 (11)	C3—Ti1—C4	34.06 (7)
C35—C31—Ti2	74.50 (11)	C14—Ti1—C4	86.87 (7)
C36—C31—Ti2	108.89 (13)	C15—Ti1—C4	88.73 (7)
C33—C32—C31	107.53 (18)	C52—Ti2—C31	75.29 (7)
C33—C32—C37	127.15 (19)	C52—Ti2—C32	107.25 (7)
C31—C32—C37	124.84 (19)	C31—Ti2—C32	35.41 (7)
C33—C32—Ti2	75.27 (12)	C52—Ti2—C35	78.80 (7)
C31—C32—Ti2	70.56 (11)	C31—Ti2—C35	35.40 (7)
C37—C32—Ti2	125.83 (14)	C32—Ti2—C35	58.40 (7)
C32—C33—C34	108.23 (18)	C52—Ti2—C43	98.29 (7)
C32—C33—C38	125.2 (2)	C31—Ti2—C43	131.46 (7)
C34—C33—C38	125.35 (19)	C32—Ti2—C43	111.23 (7)
C32—C33—Ti2	70.24 (11)	C35—Ti2—C43	166.85 (7)
C34—C33—Ti2	72.34 (11)	C52—Ti2—C42	84.79 (7)
C38—C33—Ti2	132.94 (15)	C31—Ti2—C42	153.92 (7)
C35—C34—C33	108.10 (18)	C32—Ti2—C42	146.08 (7)
C35—C34—C39	125.2 (2)	C35—Ti2—C42	154.80 (7)
C33—C34—C39	126.4 (2)	C43—Ti2—C42	34.85 (7)
C35—C34—Ti2	71.30 (11)	C52—Ti2—C41	107.63 (7)
C33—C34—Ti2	73.39 (11)	C31—Ti2—C41	170.47 (7)
C39—C34—Ti2	125.79 (15)	C32—Ti2—C41	144.62 (8)
C34—C35—C31	107.80 (19)	C35—Ti2—C41	135.44 (7)
C34—C35—C40	125.3 (2)	C43—Ti2—C41	57.70 (7)
C31—C35—C40	126.7 (2)	C42—Ti2—C41	34.67 (7)
C34—C35—Ti2	74.34 (12)	C52—Ti2—C34	111.76 (7)
C31—C35—Ti2	70.10 (11)	C31—Ti2—C34	58.04 (7)
C40—C35—Ti2	125.45 (14)	C32—Ti2—C34	57.76 (7)
C31—C36—C51	110.19 (17)	C35—Ti2—C34	34.35 (7)
C31—C36—H36A	109.6	C43—Ti2—C34	149.79 (8)
C51—C36—H36A	109.6	C42—Ti2—C34	147.36 (7)
C31—C36—H36B	109.6	C41—Ti2—C34	112.90 (7)
C51—C36—H36B	109.6	C52—Ti2—C33	131.85 (7)
H36A—C36—H36B	108.1	C31—Ti2—C33	57.80 (7)
C32—C37—H37A	109.5	C32—Ti2—C33	34.49 (7)
C32—C37—H37B	109.5	C35—Ti2—C33	57.22 (7)
H37A—C37—H37B	109.5	C43—Ti2—C33	119.85 (7)
C32—C37—H37C	109.5	C42—Ti2—C33	143.18 (7)
H37A—C37—H37C	109.5	C41—Ti2—C33	117.04 (8)
H37B—C37—H37C	109.5	C34—Ti2—C33	34.27 (7)
C33—C38—H38A	109.5	C52—Ti2—C45	140.21 (7)
C33—C38—H38B	109.5	C31—Ti2—C45	144.45 (7)
H38A—C38—H38B	109.5	C32—Ti2—C45	110.70 (7)
C33—C38—H38C	109.5	C35—Ti2—C45	132.23 (7)
H38A—C38—H38C	109.5	C43—Ti2—C45	56.92 (7)
H38B—C38—H38C	109.5	C42—Ti2—C45	56.80 (7)
C34—C39—H39A	109.5	C41—Ti2—C45	33.97 (7)
C34—C39—H39B	109.5	C34—Ti2—C45	98.43 (7)
H39A—C39—H39B	109.5	C33—Ti2—C45	87.51 (7)
C34—C39—H39C	109.5	C52—Ti2—C44	132.34 (7)
H39A—C39—H39C	109.5	C31—Ti2—C44	128.10 (7)
H39B—C39—H39C	109.5	C32—Ti2—C44	94.46 (7)
C35—C40—H40A	109.5	C35—Ti2—C44	146.15 (7)
C35—C40—H40B	109.5	C43—Ti2—C44	34.11 (7)
H40A—C40—H40B	109.5	C42—Ti2—C44	56.91 (7)
C35—C40—H40C	109.5	C41—Ti2—C44	56.83 (7)
H40A—C40—H40C	109.5	C34—Ti2—C44	115.70 (7)
H40B—C40—H40C	109.5	C33—Ti2—C44	88.93 (7)
C45—C41—C42	108.02 (17)	C45—Ti2—C44	34.01 (7)
